# The exonuclease Nibbler regulates age-associated traits and modulates piRNA length in *Drosophila*

**DOI:** 10.1111/acel.12323

**Published:** 2015-03-06

**Authors:** Virzhiniya L Feltzin, Mugdha Khaladkar, Masashi Abe, Michael Parisi, Gert-Jan Hendriks, Junhyong Kim, Nancy M Bonini

**Affiliations:** 1 Department of Biology, University of PennsylvaniaPhiladelphia, PA, 19104, USA; 2 Penn Genome Frontiers Institute, University of PennsylvaniaPhiladelphia, PA, 19104, USA

**Keywords:** aging, endo-siRNA, miRNA, Nibbler, piRNA

## Abstract

Nibbler (Nbr) is a 3′-to-5′ exonuclease that trims the 3′end of microRNAs (miRNAs) to generate different length patterns of miRNAs in *Drosophila*. Despite its effect on miRNAs, we lack knowledge of its biological significance and whether Nbr affects other classes of small RNAs such as piRNAs and endo-siRNAs. Here, we characterized the *in vivo* function of *nbr* by defining the Nbr protein expression pattern and loss-of-function effects. Nbr protein is enriched in the ovary and head. Analysis of *nbr* null animals reveals adult-stage defects that progress with age, including held-up wings, decreased locomotion, and brain vacuoles, indicative of accelerated age-associated processes upon *nbr* loss. Importantly, these effects depend on catalytic residues in the Nbr exonuclease domain, indicating that the catalytic activity is responsible for these effects. Given the impact of *nbr* on miRNAs, we also analyzed the effect of *nbr* on piRNA and endo-siRNA lengths by deep-sequence analysis of libraries from ovaries. As with miRNAs, *nbr* mutation led to longer length piRNAs – an effect that was dependent on the catalytic residues of the exonuclease domain. These analyses indicate a role of *nbr* on age-associated processes and to modulate length of multiple classes of small RNAs including miRNAs and piRNAs in *Drosophila*.

## Introduction

microRNAs (miRNAs) are 20∼24nt small RNAs that regulate diverse biological processes, such as development and age-associated diseases (Bartel, 2009; Ambros, 2011; Kato & Slack, 2012). Since the discovery of the first miRNA *lin-4* and the second miRNA *let-7* as developmental timing genes, hundreds of miRNAs have been identified across species. The canonical biogenesis pathway of animal miRNAs starts with the transcription of the primary miRNA (pri-miRNA) from the miRNA-coding genes by RNA polymerase II in the nucleus (Bartel, 2004; Czech & Hannon, 2011). Pri-miRNAs are then cleaved by two distinct RNase III/RNA-binding protein complexes. Drosha/Pasha (Drosha/DGCR8 in mammals) cleaves pri-miRNAs to generate precursor-miRNAs (pre-miRNAs) in the nucleus. After the export of pre-miRNAs to the cytoplasm by Exportin-5, Dcr-1/Loqs-PB (Dcr/TRBP in mammals) cleaves the pre-miRNAs to generate miRNA/miRNA* duplexes. One of the strands of the miRNA duplex is preferentially retained in miRISC containing Ago1 (Ago2 in mammals), which targets mRNAs through the partial complementarity between the seed sequence (nucleotides 2–8 at the 5′end of miRNA) and the mRNAs to induce translational repression and mRNA decay (Bartel, 2009; Fabian & Sonenberg, 2012).

Initial efforts to identify novel miRNAs relied on computational prediction of miRNA genes followed by Northern blots (Lai *et al*., 2003). However, development in high-throughput sequencing techniques enabled discovery of small RNA species across different biological contexts (Ruby *et al*., 2006, 2007; Landgraf *et al*., 2007; Morin *et al*., 2008; Burroughs *et al*., 2010; Westholm *et al*., 2012). The general conclusion from these profiling studies is that miRNAs are heterogeneous at the 5′end, 3′end, and even in internal sequence (Neilsen *et al*., 2012). In addition, the 3′end of miRNAs is more variable compared to the 5′end. There are a few known mechanisms by which such heterogeneity is generated. For example, Drosha and Dicer cleavages define the 5′end and 3′end of miRNAs, but the cleavages could be imprecise such that they generate 5′end and 3′end variations (Calabrese *et al*., 2007; Wyman *et al*., 2011). In line with this, the cleavage position by Dicer is controlled by its partner protein Loqs-PB in *Drosophila* and TRBP in mammals, to generate miRNA isoforms that are overlapping, but with distinct seed sequences due to a differential 5′end (Fukunaga *et al*., 2012; Lee & Doudna, 2012). In addition to Drosha and Dicer cleavages, 3′end heterogeneity of miRNAs is subject to nucleotide addition, such as adenylation and uridylation (Jones *et al*., 2009; Katoh *et al*., 2009; Burroughs *et al*., 2010). Intriguingly, the loss of such heterogeneity leads to a change in specificity and efficiency of target silencing, underscoring the functional significance of controlling end heterogeneity. Despite the accumulating evidence of miRNA heterogeneity and the identification of the enzymes to generate such heterogeneity, however, we still lack clear knowledge of the biological impact of such heterogeneity at the organismal level.

Study of *Drosophila* miR-34-5p shows that miR-34-5p displays a pattern of different length isoforms due to 3′end heterogeneity and that the short isoform accumulates with age (Liu *et al*., 2012). Loss of miR-34 leads to accelerated aging in *Drosophila*. Screening defined a 3′-to-5′ exonuclease, Nbr, which is responsible for the isoform pattern of miR-34-5p: upon knockdown of *nbr*, the long isoform accumulates, while the short isoforms decline (Han *et al*., 2011; Liu *et al*., 2011). A subset of *Drosophila* miRNAs are subject to length pattern control by Nbr; moreover, recent data have shown that the pattern may reflect differential loading into the miRISC and siRNA RISC complexes with age (Abe *et al*., 2014). Disruption of the process, by knockdown of the genes Hen1 and Ago2, leads to shorter lifespan and neurodegeneration. It is unknown whether Nbr impacts the length of classes of small RNAs beyond miRNAs, such as piRNAs and endo-siRNAs, and a detailed characterization of the *nbr* loss of function phenotype has not been performed. Here, we characterize loss-of-function effects of the *nbr* gene on the animal, and effects on piRNAs and endo-siRNAs. These analyses indicate that *nbr* loss results in accelerated age-associated defects, as well as impacting the length of piRNAs. Importantly, these effects depend on the catalytic residues of the Nbr exonuclease domain, suggesting that the processing of RNA substrates by Nbr is critical for these effects. Together, this study reveals new insight into the importance of controlling length heterogeneity of small RNAs.

## Results

### Expression pattern of Nbr *in vivo*

**Fig 1:**
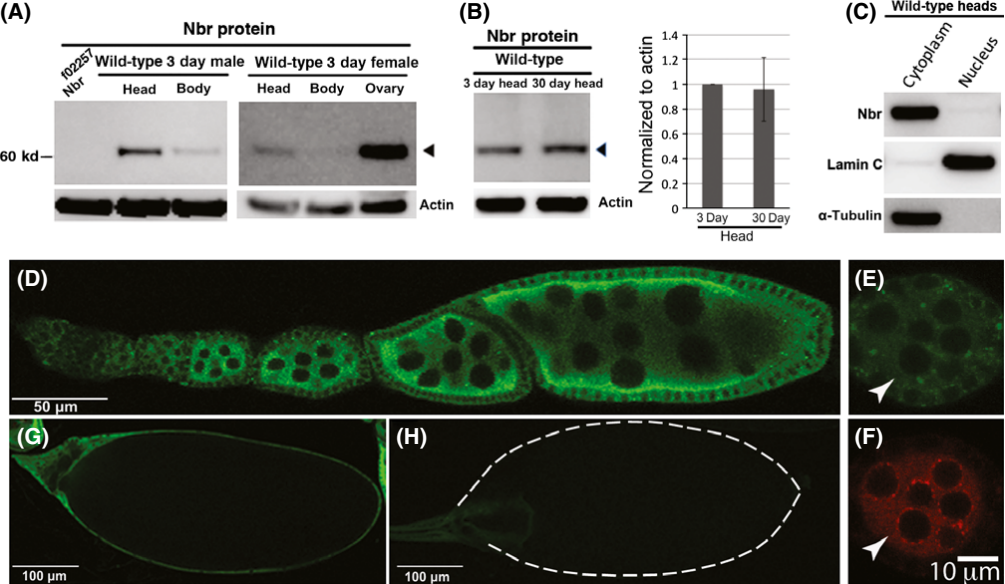
Expression pattern of the Nbr protein. (A) Western immunoblot showing Nbr protein expression in head, ovary, and body (without ovary). Left panel: Nibbler (Nbr) is enriched in the head relative to the body. Right panel: Nbr protein expression is highest in the ovary. (B) Western immunoblots of Nbr expression with age show little change between 3d and 30d. Mean±SD (*n* = 4), *P *=* *0.76 (Student's *t*-test). (C) Western immunoblot of fractionated protein from heads of controls (Bloomington line 5905) shows that Nbr protein is highly enriched in the cytoplasm (assessed by immunoblotting for α-tubulin) vs nucleus (assessed by immunoblotting for Lamin C). (D–H) Nbr expression, detected by anti-HA antibody, in confocal images from whole-mount ovaries from pCaSpeR-*nbr*.HA flies. (D) Nbr is detected throughout egg chamber development. (E, F) Expression of (E) Nbr in the germarium overlapped in the nuage (arrows) with (F) Ago3, although Nbr did not show special association with the nuage. (G) Nbr expression in stage 12 ovary. Nbr expression persists in the remnants of nurse cells, but is not detectably present in the mature oocyte. (H) Nbr is not detectable in the mature egg. The outline of the egg is shown in dashed lines.

Nibbler is a 3′-to-5′ exonuclease whose function is critical for trimming the 3′end of miR-34-5p in *Drosophila* (Han *et al*., 2011; Liu *et al*., 2011). Upon *nbr* loss, a subset of *Drosophila* miRNAs show accumulation of long isoforms. To understand the biological role of *nbr in vivo* in more detail, we first assessed the expression pattern of the Nbr protein. We developed a rabbit polyclonal antibody against the N-terminus of Nbr (see Methods). By Western immunoblot, Nbr was enriched in ovary and then heads, followed by the body (Fig.1A). Temporally, Nbr showed little change in level with age in the adult head (Fig.1B), despite the fact that the lower isoform of miR-34-5p accumulates with age (Liu *et al*., 2012). Nuclear/cytoplasmic fractionations revealed that Nbr was enriched in the cytoplasm (Fig.1C), consistent with its role in a complex with Ago1 to process the 3′end of miRNAs (Liu *et al*., 2011). To understand the spatial expression pattern of Nbr, we made a transgene containing the genomic region of *nbr* encompassing ∼650 bp upstream and ∼150 bp downstream of the open reading frame, with a 1xHA tag inserted at the C-terminus. Immunohistochemistry of the ovary with anti-HA antibody revealed that Nbr is expressed from the germarium to later stages of egg chamber development (Fig.1D). Consistent with biochemical nuclear/cytoplasmic fractionation, Nbr was enriched in the cytoplasm of nurse cells, in follicle cells, as well as present in cells of the germarium at a lower level (Fig.1D). In the germarium, Nbr was present in the cytoplasm and overlapped the pattern of Ago3 in the nuage where piRNAs are generated, although without special association (arrows, Fig.1E,F). Nbr expression remained in nurse cells until stage 11, after the nurse cells have dumped their contents into the oocyte (Fig.1G). In the oocyte and mature eggs, Nbr expression was low (Fig.1G,H). Together, these results suggest a potential importance of *nbr* in the ovary, as well as in adult tissues.

### Generation of *nbr* loss-of-function mutants

**Fig 2:**
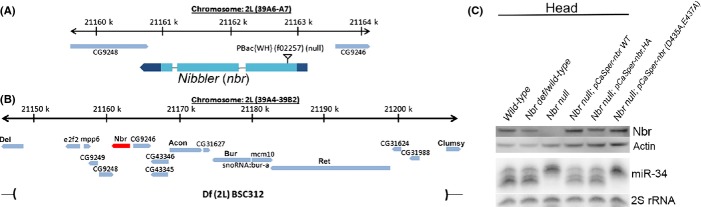
Analysis and rescue of *nbr* loss-of-function mutation. (A) Schematic of *nbr* gene chromosomal region, noting the location of the *nbr*^*f02257*^ allele Piggybac transposon (see Liu *et al*., 2012). (B) Chromosome schematic of the deficiency used *in trans* to *nbr*^*f02257*^ allele to generate *nbr* null activity. Deleted region is indicated by parentheses (see Flybase GBrowse). (C) Nbr protein level and miR-34 pattern in various strains. Western immunoblots for Nbr show that protein is not detectable in *nbr* null (*nbr*^*f02257*^*/Df(2L)BSC312*) flies in heads; Nbr protein level is recovered in lines pCaSpeR-*nbr* (WT) and pCaSpeR-*nbr*.HA, and catalytically dead pCaSpeR-*nbr* (D435A,E437A). Immunoblot for Actin shows all lanes have protein. Small RNA Northerns show that the miR-34 pattern is compromised in *nbr* null. However, the miR-34 pattern is rescued by pCaSpeR-*nbr* (WT) and pCaSpeR-*nbr*.HA transgenes. The genomic rescue construct expressing catalytically dead Nbr failed to rescue the miR-34 isoform pattern, despite robust levels of the Nbr protein. 2S rRNA, loading control for Northerns. Genotypes in C are as follows: wild-type is *w*^*1118*^. *nbr* def/wild-type is *Df(3L)BSC312*/+. *nbr* null is *nbr*^*f02257*^*/Df(3L)BSC312*. *nbr* null, pCaSpeR-nbr WT is *nbr*^*f02257*^*/Df(3L)BSC312; pCasper-nbr WT/+*. *nbr* null, pCaSpeR-nbr.HA is *nbr*^*f02257*^*/Df(3L)BSC312; pCaSpeR-nbr-HA/+. nbr null, pCaSpeR-nbr (D435A.E437A)* is *nbr*^*f02257*^*/Df(3L)BSC312; pCasper-nbr(D435.E437A)/+*.

Previous studies suggested lethality and sterility upon *nbr* knockdown (Han *et al*., 2011; Liu *et al*., 2011). To rigorously examine whether these effects were indeed associated with loss of *nbr* function, we crossed the *nbr*^*f02257*^ allele to a deficiency line (*Df(2L)BSC312*) that deletes a ∼60 kb region that includes the *nbr* gene (Fig.2A,B). We refer to the *nbr*^*f02257*^*/Df(2L)BSC312* heterozygotes as ‘*nbr* null’ because we cannot detect the Nbr protein (Fig.2C), although we cannot rule out that some level of protein is expressed that is below the level of detection in our assays. We found that this allelic combination was viable and fertile. Thus, the previously observed lethality and sterility appear unlinked to *nbr* (Han *et al*., 2011; Liu *et al*., 2011). miR-34-5p trimming was compromised in the *nbr* null combination, and the Nbr protein level and miR-34-5p trimming pattern were fully rescued by introducing the wild-type genomic *nbr* transgene (Fig.2C). Importantly, rescue of miRNA trimming activity was dependent on the catalytic residues of the Nbr exonuclease domain: mutating catalytic residues (D435A,E437A) within the genomic transgene (referred to as ‘cat-dead *nbr*’) failed to rescue miR-34-5p trimming, despite robust protein expression (Fig.2C). These data indicate that the lethality and sterility effects, previously thought to be associated with *nbr*, are instead independent of *nbr* function, and rather that the *nbr* null genetic combination yields viable and fertile adults.

### *nbr* mutants show accelerated age-associated effects

Using the *nbr* null chromosomal combination (*nbr*^*f02257*^*/Df(2L)BSC312*), we assessed the effect of the loss of *nbr* function to the animal. Although Nbr was highly expressed in the ovary, we saw no effect on ovary development in *nbr* null animals (Fig. S1A) and were able to obtain viable eggs from *nbr* null females. In addition, the number of eggs laid per day did not differ significantly between *nbr* null and *nbr* wild-type rescued females with age (Fig. S1B).

**Fig 3:**
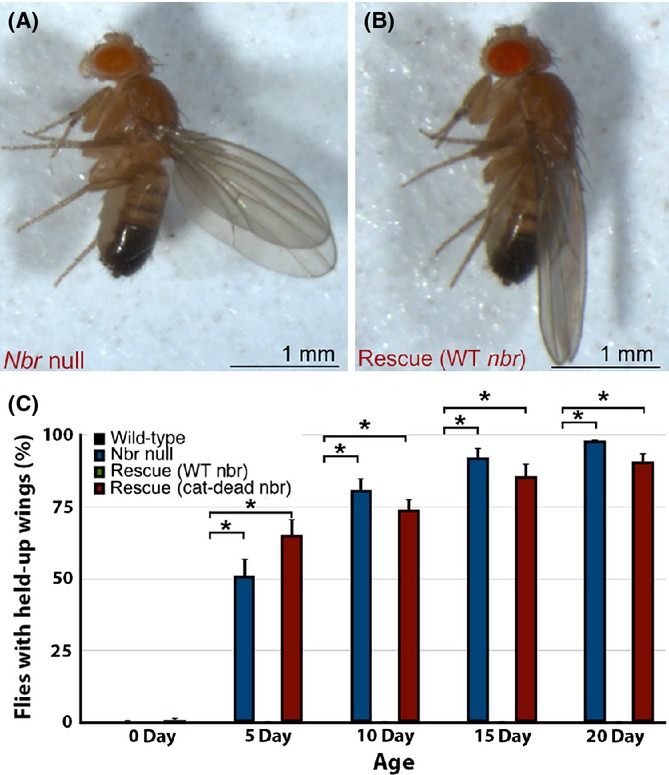
*nbr* null (*nbr*^*f02257*^*/Df(3L)BSC312*) animals have held-up wings. (A,B) Images to illustrate normal wing posture compared to held-up wings. Adult male flies at 14d. (A) *nbr* null (*nbr*^*f02257*^*/Df(3L)BSC312*) animals have a held-up wing posture. (B) *nbr* null; pCaSpeR-*nbr* (wild-type) flies (‘rescue (WT *nbr*)’) are rescued to normal wing posture. (C) Quantification of flies with normal wing posture with age. Wing posture was scored on awake, unanesthetized animals. Wild-type and rescued flies have normal wing posture, whereas *nbr* null and *nbr* null animals rescued with a catalytically dead *nbr* construct show a progressive held-up wing posture. Values are mean ± standard error of three independent experiments. *P < 0.01 compared to wild-type control using one-way anova followed by Dunnett's test.

We then examined the adult animal for effects. Intriguingly, *nbr* null animals showed an age-associated held-up wing phenotype (Fig.3). At the time of emergence of the adult (eclosion), *nbr* null animals showed normal wing posture. However, by 20d, >90% of *nbr* mutants showed held-up wings (Fig.3C). This effect was fully rescued by wild-type, but not the cat-dead *nbr* genomic transgene, indicating that the exonuclease activity of Nbr was required for normal wing posture. Whether this effect was due to a problem in muscle or nerve, or both, is an interesting question; however, our attempts to map this wing effect to a tissue were unsuccessful due to leakiness of our transgenes expressing Nbr, regardless of the presence of a GAL4 tissue-specific driver (M. Abe and N. M. Bonini, unpublished data).

**Fig 4:**
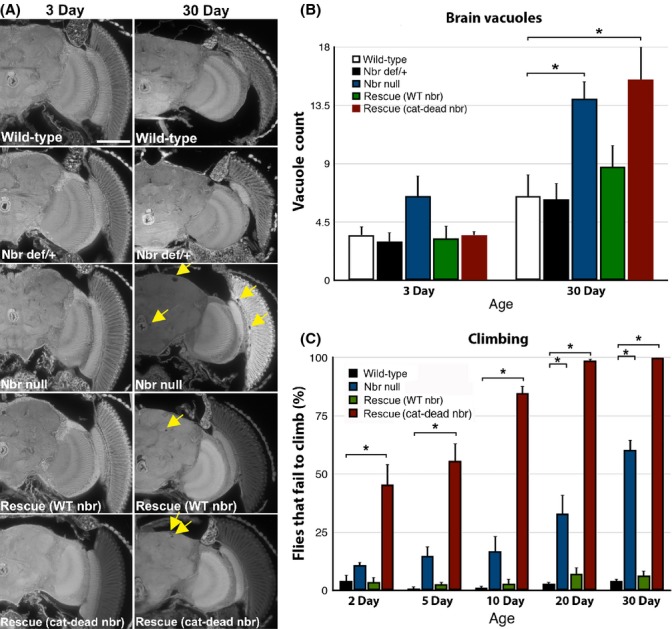
*nbr* null animals show age-associated defects. Paraffin sections of wild-type, *nbr* def/+ (*Df(3L)BSC312*/+), *nbr* null (*nbr*^*f02257*^*/Df(3L)BSC312*)*,* rescue (WT *nbr*) (*nbr* null; pCaSpeR-*nbr* (wild-type)/+), and rescue (cat-dead *nbr*) (*nbr* null; pCaSpeR-*nbr* (D435A,E437A)/+) heads at 3d and 30d. Scale bar in top left panel, 0.1 mm for all panels. (B) Quantification of brain vacuolization at 3d and 30d. *nbr* null shows an increase in vacuoles with age that is rescued by WT *nbr* transgene but not cat-dead *nbr*. (C) Climbing ability of animals with age shows that *nbr* null displays rapid loss of climbing that is rescued by WT *nbr*, but is worse with cat-dead *nbr*. Values in B and C are mean ± standard error of the mean of three independent experiments. *P < 0.01 compared to wild-type control using one-way anova followed by Dunnett's test.

Examination of *nbr* null animals for other effects revealed additional age-associated deleterious phenotypes of brain degeneration and loss of climbing ability. Nbr mutants showed no brain vacuolization as young animals (3d), but a high level of brain vacuoles by 30d (Fig.4A,B). This effect was rescued by expression of the normal *nbr* genomic transgene, but not by the cat-dead *nbr* genomic transgene. In addition, *nbr* mutants showed dramatic loss of climbing ability: while wild-type animals show little change in climbing ability even at 30d (>90% of wild-type flies successfully climbed at 30d), *nbr* null animals showed a dramatic decline already by 20d (∼50% of flies failed to climb) (Fig.4C). This effect was also fully rescued by the wild-type, but not cat-dead, *nbr* genomic transgenes (Fig.4C). Together, these results suggest that *nbr* loss is associated with early onset of several age-associated effects in *Drosophila*.

### Nbr affects the length of piRNAs

Nbr has been shown to impact the length of a subset of miRNAs in *Drosophila* (Han *et al*., 2011; Liu *et al*., 2011). However, it is unknown whether other classes of small RNAs, such as Piwi-interacting RNAs (piRNAs) (Guzzardo *et al*., 2013) and endogenous siRNAs (endo-siRNAs) (Czech & Hannon, 2011), are also affected by *nbr*. piRNAs are ∼24-30nt small RNAs that are bound to Piwi-clade proteins of the Argonaute family, and are derived from heterochromatin, 3′UTRs of protein-coding genes, and euchromatic transposable elements. Endogenous siRNAs are ∼21nt small RNAs identified in *Drosophila*, as well as *C. elegans* and mammals (Czech & Hannon, 2011). In *Drosophila,* endo-siRNAs are bound to Ago2, an siRISC component. Like piRNAs, endo-siRNAs are also derived from transposable elements, as well as other overlapping and hairpin RNA sources. Both piRNAs and endo-siRNAs are suggested to silence transposable elements, indicating the importance of these two classes of small RNAs to maintain genome integrity in the animal.

Given the high expression level of Nbr in the ovary, we examined the effect of *nbr* loss of function on piRNA and endo-siRNA length in ovary libraries. We deep-sequenced and analyzed small RNA libraries from ovaries of wild-type, *nbr* def/+ (*Df(2L)BSC312*/+), *nbr* null, *nbr* null with pCaSpeR-*nbr* (WT) (‘rescue (WT *nbr*)’), and *nbr* null with pCaSpeR-*nbr* (D435A,E437A) (‘rescue (cat-dead *nbr*)’). Because none of these genotypes had defects in ovary development (see Fig. S1 and data not shown), none of the effects on the length of endo-siRNAs and piRNAs were likely due to loss of specific tissue or cell types in the ovary.

**Fig 5:**
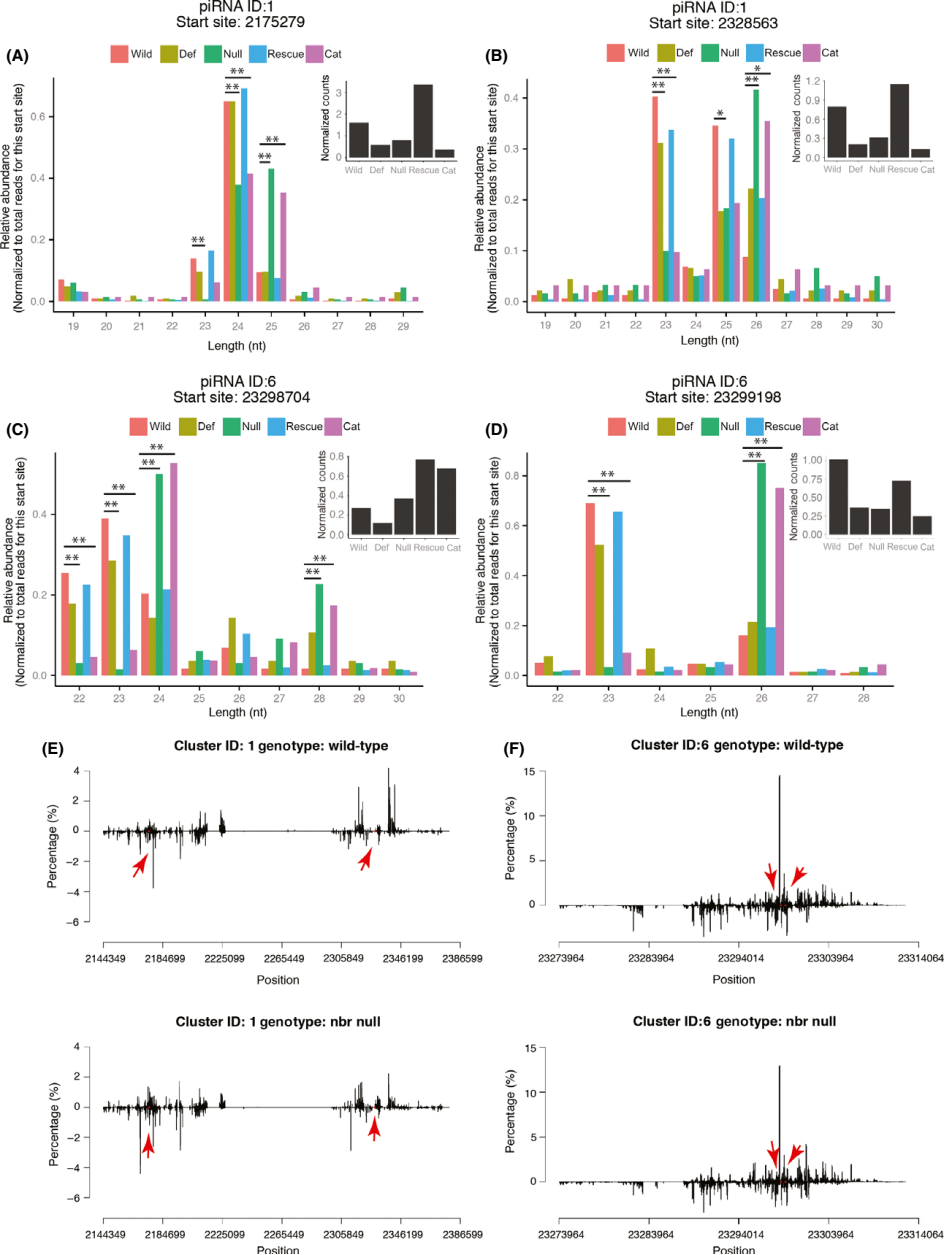
*nbr* affects the length of piRNAs with the same 5′end. A–D. Abundance of piRNA reads with the indicated length relative to the total number of reads originating from that specific piRNA locus and start site. Graphs for piRNA locus 1, start sites (A) 2175279 and (B) 2328563, and piRNA locus 6, start sites (C) 23298704 and (D) 23299198, are highlighted with red dots and arrows in E and F. Insert shows abundance of the specific reads plotted, which is not consistently different for the different genotypes. Start sites are numbered according to the position of the first nucleotide of the reads in the reference chromosome sequence (*dm3 Drosophila* genome assembly). E,F. Density plots of piRNA loci 1 (E) and 6 (F) for wild-type (top panels) and *nbr* null (bottom panels) flies. We observed no significant difference in genomic location of origin or overall read abundance of piRNA loci between the genotypes. The start positions for the piRNA reads that are of different length in *nbr* vs control in A–D are noted with red dots and highlighted with red arrows. Genotypes: wild, wild-type (*w*^*1118*^). def: *nbr* deficiency/+ (*Df(3L)BSC312*/+). null: *nbr* null (*nbr*^*f02257*^*/Df(3L)BSC312*). rescue: *nbr* null with genomic rescue of wild-type *nbr* (*nbr* null; pCaSpeR-*nbr* (wild-type)/+). cat: *nbr* null with genomic rescue with catalytically dead *nbr* (*nbr* null; pCaSpeR-*nbr* (D435A,E437A)/+). The t-statistic describing the ratio of departure in the abundance of piRNA reads compared to the wild-type control was calculated for each specific piRNA locus, start site, and read length. The t-statistic was then converted to the corresponding *P*-value using the sum total of reads being considered as the number of degrees of freedom, and it was adjusted for multiple comparisons using the false discovery rate (≤0.05). In addition, we computed Cohen's d to indicate the effect size. *P < 0.05 compared to wild-type and Cohen's d ≥ 0.6. **P < 0.01 compared to wild-type and Cohen's d ≥ 0.6. Detailed information on all piRNAs with significance is listed in Table S1 (Supporting information).

We first confirmed the effects of Nbr on miRNAs, such as miR-34-5p and miR-305-5p in the ovary libraries. These miRNAs showed a higher portion of longer isoforms in the *nbr* null, which was rescued by the wild-type, but not by the cat-dead *nbr* transgene (Fig. S2A–D). We also confirmed the effect of Nbr on other known Nbr-dependent miRNAs (Fig. S2E, red diamonds). We then mapped non-miRNA reads onto identified piRNA (Brennecke *et al*., 2007) and endo-siRNA (Czech *et al*., 2008) loci. There was no change in overall read abundance for any piRNA locus. However, *nbr* loss of function led to a statistically significant increase in piRNA length for a specific subset of piRNAs, defined by their 5′end position. This effect was dependent on *nbr* catalytic activity. Among the 142 piRNA clusters examined (Brennecke *et al*., 2007), 138 loci were expressed in all of our genotypes with ≥5 reads in at least one of the genotypes. For each piRNA locus and for each piRNA 5′ origin position within the locus, we quantified the fraction of reads corresponding to the different lengths in each of the genotypes and analyzed them for modulation by *nbr* gene function. Among these 138 piRNA loci, we found 48 loci (35%) were significantly affected by *nbr* loss in at least one start position within the locus: longer piRNAs accumulated, while shorter piRNAs decreased (Fig.5, Table S1). This trend was rescued by wild-type, but not by the cat-dead, *nbr* genomic transgenes. Thus, while there was no dramatic effect on the generation of piRNAs, *nbr* function appeared to modulate the length of about half of the piRNAs, by shifting the length toward longer length piRNAs that start at any one 5′end position. Attempts to verify length changes by Northern blot were unsuccessful; elements detected by piRNAs are redundant, complicating the ability to see small changes in any one locus.

piRNAs are predominantly derived from germline cells and associated with Aub, Ago3, and Piwi (Brennecke *et al*., 2007). However, piRNAs are also expressed in somatic cells of the ovary, which are predominantly derived from the flamenco locus (Malone *et al*., 2009). To examine whether the effect of Nbr on piRNAs was tissue specific, we mapped small RNA reads to the flamenco locus as well. This analysis indicated that Nbr loss did not lead to accumulation of long forms of flamenco piRNAs (data not shown). Thus, Nbr predominantly affects the length of piRNAs from germline cells and not somatic cells of the ovary of *Drosophila*.

We next examined individual endo-siRNA loci. Among the 49 endo-siRNA loci identified in *Drosophila* ovaries (Czech *et al*., 2008), none of the loci appeared to be modulated by *nbr* as defined by our statistical threshold (see Methods).

## Discussion

Here, we have characterized the biological function of the *nbr* gene to the organism in *Drosophila* and found that loss of *nbr* leads to age-associated defects of brain degeneration, declining locomotion, and wing postural defects. Although previous studies suggested lethality and sterility were associated with *nbr*, in the studies here we used a combination of the original *nbr* allele in *trans* to a deficiency for the locus. In addition, we rescued the effects of this *nbr* null combination with genomic *nbr* expression transgene, but not by a catalytically dead transgene, confirming association with *nbr* function, and indicating that these defects are indeed associated with the enzymatic activity of Nbr. Some of these defects associated with *nbr* overlap with those of miR-34-5p (brain degeneration, loss of climbing ability, shorter lifespan); however, whether these phenotypes depend on the loss of 3′end trimming of miRNAs and notably miR-34-5p, or other functions of Nbr on small RNAs, awaits further study. We also observed that the *nbr* null animals rescued with the catalytically mutated genomic *nbr* transgene showed a consistent and striking lifespan reduction of ∼50% (M. Abe and N. M. Bonini, unpublished data), suggesting potentially broader roles of Nbr. We note that this effect and the effect on climbing (see Fig4C) are worse with the catalytically dead genomic transgene compared to the null situation. This indicates that there may be residual *nbr* activity for those functions that is worse with the catalytically dead form or that *nbr* functions in a complex with a more severe effect when a catalytically compromised protein is expressed.

Given that Nbr affects the length of not only miRNAs, but also piRNAs, Nbr could potentially trim the 3′ends of a much broader species of RNA substrates, including other long and short noncoding RNAs and mRNAs. It is worth-noting that the potential homologues of the Nbr exonuclease domain in mammals include EXD3 and Werner syndrome protein (WRN) (Liu *et al*., 2011), the latter of which is associated with a premature aging disorder. Even though it is not yet clear whether mammalian WRN impacts 3′end heterogeneity of small RNAs, future studies in mammalian systems might reveal a link between the control of 3′end heterogeneity of small RNAs and aging processes across species.

Ours and others’ studies suggest a model of how Nbr impacts the length of miRNAs (Han *et al*., 2011; Liu *et al*., 2011). In this model, Nbr interacts with Ago1 (miRISC) to trim the 3′end of miRNAs. The findings we present here indicate that Nbr also impacts the length of select piRNAs, leading to their shortening at the 3′end. The current model for piRNA biogenesis suggests that the primary biogenesis starts with the transcription of the piRNA clusters (Guzzardo *et al*., 2013). The 5′end of piRNAs is thought to be defined by an endonuclease Zucchini (Zuc) (Ipsaro *et al*., 2012; Nishimasu *et al*., 2012). Following the loading of the piRNA intermediates into Piwi proteins, a Mg^2+^-dependent 3′ trimming activity is required to generate mature lengths of piRNAs (silkworm *in vitro* assay (Kawaoka *et al*., 2011)).

Based on this model, one possibility is that Nbr, a predicted Mg^2+^-dependent 3′-to-5′ exonuclease, might trim the end of piRNA intermediates to generate different length piRNAs. Our finding that loss of *nbr* gene function affects the length of piRNA reads with the same 5′ start site is consistent with a model in which trimming of the piRNA 3′end occurs after the 5′end has been defined. In considering this model, it is of note that piRNAs and endo-siRNAs are protected at the 3′end by 2′-*O-*methylation, in contrast to miRNAs that are typically unmodified at the 3′end (Czech & Hannon, 2011). Given that 2′-*O-*methylation of small RNAs at the 3′end is protective from 3′end trimming, by this model, Nbr likely trims the 3′end of piRNAs prior to the protection by 2′-*O-*methylation. Alternatively, Nbr might affect the length of piRNAs in a more indirect manner. It is intriguing that the three *Drosophila* Piwi-clade proteins, Piwi, Aub, and Ago3, are loaded with slightly different length piRNAs: Piwi is bound by the longest piRNAs, followed by Aub and Ago3 (Brennecke *et al*., 2007). Given this observation, the change in the length of piRNAs upon *nbr* loss might instead reflect a change in the loading pattern of piRNAs among these different Piwi-clade proteins. Further studies are required to reveal additional mechanistic detail into *nbr* modulation of the length of piRNAs.

Despite the effect of *nbr* on the length of piRNAs, *nbr* loss did not lead to an obvious developmental defect in the ovary or a change in fertility with age, in contrast to other mutations with severe effects on piRNA biogenesis (Lin & Spradling, 1997; Cox *et al*., 1998; Harris & Macdonald, 2001). Rather, our analysis indicates that Nbr has a subtle impact on shaping the piRNA population. This fine-tuned role of *nbr* explains why screens for components of the primary piRNA biogenesis pathway did not identify *nbr* as a candidate (Czech *et al*., 2013; Handler *et al*., 2013; Muerdter *et al*., 2013), as such screens have been focused on genes with dramatic effects. The role of trimming by Nbr on miRNA function has been assessed with the study of miR-34 upon expression of a catalytically mutant form of *nbr* in cultured cells, where miR-34 silencing was compromised by ∼20% (Han *et al*., 2011). The functional impact of *nbr* on piRNA length for their activity may also be similarly fine-tuned; our attempts to define an impact on transposon activity in the ovary failed to reveal an effect (M. Abe and N. M. Bonini, unpublished data). However, it is intriguing that transposons have recently been shown to become mobilized in the adult fly brain with age (Li *et al*., 2013; Perrat *et al*., 2013), and our other studies have indicated a shift with age in the loading of miRNAs between the miRNA RISC and the siRNA RISC (Abe *et al*., 2014), coupled with age-associated defects at the organismal level if the integrity of small RNAs that are loaded into siRISC is compromised. Given the impact of *nbr* on age-associated biological processes and the modulation of small RNA loading with age revealed by our earlier study of trimmed RNAs (Abe *et al*., 2014), there may be additional roles of *nbr* on small RNA populations in the brain with age.

## Materials and methods

### Fly stocks

Flies were grown in standard cornmeal molasses agar medium at 25 °C. *nbr*^*f02257*^ (FlyBase ID: FBti0050491) and *nbr* deficiency line (*Df(2L)BSC312*/CyO) (FlyBase ID: FBst0024338) were obtained from the Bloomington Stock Center. *nbr*^*f02257*^ line was backcrossed into a homogenous wild-type background (Bloomington Stock Line 5905 (BL5905), FlyBaseID: FBst0005905, *w*^*1118*^) for five generations. All lines used were generated or crossed into the same wild-type background, to maintain a homogenous and consistent background for all assays.

### Genomic *nbr* transgenic flies

Wild-type, HA-tagged (C-terminus of Nbr), and the catalytically mutated (D435A,E437A) *nbr* transgenes, which include ∼650 bp upstream and ∼150 bp downstream of *nbr* ORF, were generated by PCR and cloned into pCaSpeR4 vector. Because of unanticipated difficulties on amplifying the entire region of *nbr* locus with a single PCR reaction, we amplified the genomic *nbr* region with the combination of regular PCR from genomic DNA (BL 5905) and overlapping PCR to eventually amplify the whole *nbr* genomic region (∼3.2 kb) using the primers, 5′-TAAGAAGCGATCTGCGGAAT-3′ and 5′-TGCGGTTTCGTCTTCCTGAT-3′. The concept of overlapping PCR is described in Young & Dong (2004) and Xiong *et al*. (2006). Based on this wild-type genomic *nbr* sequence, we inserted 1 ×  HA tag sequence at the C-terminus, as well as inserted mutations (D435A,E437A) in the exonuclease domain by additional PCRs. The genes were then subcloned into pCaSpeR4. Constructs were sequence-verified. Genomic constructs were prepared by maxi-prep (Qiagen, Germantown, MD, USA) and injected into BL5905 (*w*^*1118*^) (Genetic Services, Cambridge, MA, USA), and transgenic flies were selected and maintained over the TM6C,Sb balancer chromosome.

### Nbr antibody

A rabbit polyclonal antibody against the Nbr protein was developed using a synthetic peptide of residues 10-23 (C-AIPAGFESDEENME) (ProSci Incorporated, Poway, CA, USA). The serum was affinity-purified to enrich for IgG directed to the Nbr peptide antigen. For Western immunoblots, the purified antibody was used at 1/2000.

### Western immunoblots

Fly tissues were resuspended in RIPA buffer, followed by grinding and centrifugation to remove debris. The supernatant was measured by Bradford assay, and 25–50 μg of protein in NuPAGE LDS sample buffer (4x) (NP0007, Life Technologies, Carlsbad, CA, USA) was loaded in each lane. NuPAGE Novex 4-12% Bis-Tris gel (#NP0321BOX, Life Technologies, Carlsbad, CA, USA) were loaded and run in 1xNuPAGE MES SDS Running Buffer (#NP0002, Life Technologies, Carlsbad, CA, USA). Western transfer was performed on PVDF membrane, followed by blocking in 5% milk/TBST for 1 h at 4 °C. The membrane was incubated with primary antibody (anti-Nbr, 1/2000) at 4 °C overnight. After washing the membrane in TBST buffer 3 times (5 min each), the membrane was incubated with secondary antibody (goat anti-rabbit IgG-HRP, #sc-2030, Santa Cruz Biotechnology, Inc., Dallas, TX, USA) at 4 °C for 2 h. The membrane was washed in TBST 3 times (5 min each), followed by signal development by Pierce ECL plus Western Blotting Substrate (#32132; Thermo Fisher Scientific, Waltham, MA, USA). The image was scanned by Fujifilm LAS-3000 Imager (Fujifilm, Tokyo, Japan).

### Immunohistochemistry

Ovaries were dissected in PBS, followed by fixation in 4% paraformaldehyde in PBS for 20 min at room temperature. After washing with PBST (PBS + 0.2% Triton X-100) twice, the ovaries were permeabilized in PBST-5 (PBS + 0.5% Triton X-100 with 5% goat serum). After washing once with PBST-B (PBS + 0.2% Triton X-100 + 1% BSA), the ovaries were incubated with primary antibody (1:150, rat monoclonal anti-HA: 3F10, #11867423001, Roche, Indianapolis, IN, USA and rabbit anti-Ago3 (1:1000) (Brennecke *et al*., 2007)) in PBST-B at 4 °C overnight. Washing was performed in PBST 3 times, 2 min each at room temperature, followed by 20 min wash in PBST 3 times and 2 min wash in PBST-B once at room temperature. The ovaries were incubated with secondary antibody Alexa Fluor 488 goat anti-rat IgG (H+L) (1:100, #A-11006, Life Technologies) in PBST for 2 h at 4 °C. After secondary antibody incubation, washing was performed in PBST three times, 2 min each at room temperature, followed by 20 min wash in PBST three times and 2 min wash in PBST-B once at room temperature. After washing, the ovaries were carefully separated on slides by fine forceps and mounted with Vectashield mounting medium with DAPI (#H-1200; Vector laboratories, Burlingame, CA, USA).

### Small RNA Northern hybridization

Total RNA was extracted from fly tissues using Trizol Reagent (#15596-018, Life Technologies), following the manufacturer's protocol. Total RNA of 3–10 μg depending on the experiment were loaded per lane onto 15% TBE-urea gels (#EC6885BOX, Life Technologies Carlsbad, CA, USA), and run for 90 min at 180 V, followed by the transferring to nylon membrane (Hybond N+, GE Healthcare, Piscataway, NJ, USA). After UV cross-linking and prehybridization (50 °C, 1 h), the membranes were hybridized with P^32^-labeled probes overnight at 50 °C. Two DNA oligonucleotides were annealed to obtain the template for each RNA probe. The DNA oligonucleotides used to make probe templates were miR-34-5p (5′-GAT AAT ACG ACT CAC TAT AGG GAG A-3′/5′-AAA AAA TGG CAG TGT GGT TAG CTG GTT GTG TCT CCC TAT AGT GAG TCG TAT TAT C-3′) and 2S rRNA (5′- GAT AAT ACG ACT CAC TAT AGG GAG A-3′/5′-TGC TTG GAC TAC ATA TGG TTG AGG GTT GTA TCT CCC TAT AGT GAG TCG TAT TAT C-3′). Probe syntheses were performed by *in vitro* transcription using MAXIscript T7 Kit (#AM1312, Life Technologies, Carlsbad, CA, USA), supplemented with P^32^-α-UTP.

### Small RNA deep sequencing

Ovaries were dissected in PBS, and 40ug total RNA was prepared from 4 to 7d ovaries of wild-type, *Df(2L)BSC312*/+, *nbr* null (*nbr*^*f02257*^*/Df(2L)BSC312*), wild-type rescue (*nbr* null; pCaSpeR-*nbr* (WT)), and catalytically dead rescue (*nbr* null; pCaSpeR-*nbr* (D435A,E437A)), using Trizol Reagent (#15596-018, Life Technologies Carlsbad, CA, USA) following the manufacturer's protocol. The small RNAs between ∼16 and ∼29 nt in size were purified from 15% TBE-urea gel (#EC6885BOX, Life Technologies Carlsbad, CA, USA). Small RNA libraries were prepared using Illumina's TruSeq small RNA sample preparation kit (#RS-200-0012, Illumina, Inc. San Diego, CA, USA), following the manufacturer's protocol. The libraries were sequenced on HiSeq2000 platform (Illumina, San Diego, CA, USA).

### Held-up wing analysis

Adult male flies were collected on the day of eclosion, aged at 25 °C with 15 flies per vial, and transferred to new fly food vials every other day. At each time point, the number of flies with held-up wings was scored. Wing posture was scored on awake, unanesthetized animals. Thirty flies were used for each genotype/time point, and the average of three independent experiments was calculated.

### Climbing assay

Adult male flies were collected on the day of eclosion, aged at 25 °C with 15 flies per vial, and transferred to new fly food vials every other day. For the climbing assay, flies aged to specific time points were incubated in the dark at room temperature for 30 min, followed by transferring into 14-ml polystyrene round-bottom vial (Falcon). Under red light, flies were gently banged to the bottom, and the percentage of flies that successfully climbed higher than 2.5 cm from the bottom of the vial in 10 s was recorded. Three scorings were performed for each genotype/time point in each biological replicate, and the average was calculated. Approximately 30 flies for each genotype/time point were used for each experiment. Three independent experiments were performed in total.

### Brain paraffin sections

Adult female heads (3d and 30d) were used for paraffin sections as described (Liu *et al*., 2012; Abe *et al*., 2014). Brain vacuoles were counted in the whole area of each brain section except retina, through five continuous frontal sections, starting from the center section (defined as the section in which the brain occupies the largest region, and the esophagus is obvious) toward posterior direction.

### Computational analyses

#### Alignment

Trimming of the adapter sequences from the obtained reads was performed by an in-house script (S. Fisher and J. Kim). The trimmed reads were then aligned to the *dm3* (BDGP Release 5) genome assembly of *Drosophila melanogaster* using the RUM pipeline (Grant *et al*., 2011). We set RUM to use 0 mismatches while aligning using Bowtie (Langmead, 2010) and 100% identity for Blat (Kent, 2002). GEO number for libraries is GSE50055.

#### Mapping to small RNAs

miRNA stem-loop sequences were obtained from miRBase. Genome coordinates of piRNA loci in *Drosophila* ovaries were obtained from Brennecke *et al*. (2007). Endogenous siRNA loci in *Drosophila* ovaries were obtained from Czech *et al*. (2008). Only the uniquely mapped reads with no mismatches and length ranging from 19 to 31 nt that mapped to each small RNA locus were counted. To control for sequencing depth, the read counts for each locus were normalized by dividing them with the total small RNA read number in each library after trimming. In addition to enumerating the total number of reads mapping to each small RNA locus, we also quantified the read counts for each unique isoform.

#### Length distribution and density plot of piRNAs and endo-siRNAs

We only considered the small RNA loci that were expressed in all of the five genotypes and comprised of reads belonging to two or more length categories (19–31 nt) with a total read count of ≥5 in at least one of the genotypes. Then for each of the genotypes, for each small RNA locus, and for each origin position within that locus, we computed the *t*-statistic for every length category present to measure the departure of the fraction of reads of a given length from that of wild-type. The *t*-statistic is the ratio of the difference between the observed value and the expected value to the standard error. The *t*-statistic was converted to the *P*-value, and we then identified significant upward and downward peaks in the distribution (FDR ≤ 0.05 and effect size indicated by Cohen's d ≥ 0.6 which corresponds to a moderate-to-large effect (Cohen, 1988)). Small RNAs whose long isoforms significantly increased in abundance in *nbr* null and catalytically dead *nbr* rescue genotypes compared to wild-type control, and whose long isoforms did not increase significantly in *nbr* def/+ and *nbr* wild-type rescue genotypes compared to wild-type control, were considered to be affected by *nbr*. A detailed list of start positions, the effect of increase length or decreased length, Cohen's d value, and *P*-values are given in Table S1 (Supporting information).

#### miRNA ratio plot

For generating miRNA ratio plot, the most abundant isoform from each 5p and 3p arm of each miRNA stem-loop was first identified. The fraction of the most abundant isoform among all the isoforms starting with the same 5′ nt position was calculated in wild-type and *nbr* null. Finally, the ratio of the wild-type and *nbr* null fractions was calculated by dividing *nbr* null/wild-type fraction (‘Ratio *nbr* null/wild-type’, *y*-axis of Figure S2). Only miRNA isoforms with > 70 raw reads in both wild-type and *nbr* null are shown in the graph (Figure S2).

### Egg-lay count

The effect of *nbr* loss-of-function mutation versus *nbr* wild-type rescue on female fecundity was examined by counting eggs laid each day by single females over a 40-day time course. A standard cornmeal/molasses medium was poured into lids of 35 × 10 mm tissue culture dishes which served as egg-laying plates that were taped to the bottom of cages made from *Drosophila* culture bottles with multiple small holes punched in to provide ventilation. Single females of the *nbr* null genotype (*nbr*^*f02257*^/*Df(2L)BSC312*) and *nbr* wild-type rescue (*nbr*^*f02257*^/*Df(2L)BSC312*; pCaSpeR-*nbr*) were placed in cages with the plates sprinkled with active yeast to promote egg laying. The plates were replaced every day and eggs laid over 24-h periods were counted.

## Supporting Information

**Table S1.** piRNA lengths affect by *nbr*.

**Fig. S1.** Nbr null females have normal ovaries and normal fecundity with age. **Fig. S2.** Length distribution of select miRNAs in ovary deep-sequencing libraries.
