# Supplementary material for: The exonuclease Nibbler regulates age-associated traits and modulates piRNA length in Drosophila
Source: Aging Cell. 2015 Mar 6;14(3):443–52. doi: 10.1111/acel.12323 (PMC4406673; doi:10.1111/acel.12323)
Supplement: Supplementary file 1 [file acel0014-0443-sd1.pdf]

Supplementary Table S1: piRNA lengths affect by *nbr*

piRNA locus ID	Transcript start position	Read Length	Change in *nbr* null compared to *wildtype*	Cohen's d	FDR
1	2154077	25	downregulated	1.658180907	3.53E-46
1	2154077	26	upregulated	1.550761643	1.09E-41
1	2154469	26	downregulated	0.848508929	6.30E-07
1	2154469	27	upregulated	1.051022935	7.40E-10
1	2161983	23	downregulated	0.863073366	1.47E-09
1	2161983	26	upregulated	0.731519507	2.97E-07
1	2162127	23	downregulated	1.831036478	3.30E-40
1	2162127	26	upregulated	1.344890451	5.62E-25
1	2162645	26	downregulated	0.677819926	0.001257437
1	2162645	28	upregulated	0.741829514	0.000394369
1	2168477	25	downregulated	0.667466017	1.57E-05
1	2168477	29	upregulated	0.703821961	4.71E-06
1	2169211	22	downregulated	0.644926594	0.018233957
1	2169211	24	downregulated	2.264433813	2.44E-14
1	2169211	25	upregulated	1.957392006	8.15E-12
1	2169220	24	downregulated	0.948372579	1.33E-08
1	2169220	27	upregulated	0.945073168	1.50E-08
1	2169394	26	downregulated	0.843078198	0.002035751
1	2169394	28	upregulated	0.662670129	0.024706361
1	2172021	19	downregulated	1.025885487	0.007145155
1	2172021	20	downregulated	1.166816877	0.00400912
1	2172021	27	upregulated	1.443988793	0.000279717
1	2173551	24	downregulated	0.632823571	3.80E-08
1	2173551	26	upregulated	0.755894836	3.85E-11
1	2174336	25	downregulated	3.386509578	3.24E-11
1	2174336	26	upregulated	2.308830341	2.54E-07
1	2174367	27	downregulated	1.579759982	8.27E-11
1	2174367	29	upregulated	1.495524221	5.79E-10
1	2174394	25	downregulated	1.150868919	0.00105919
1	2174394	26	upregulated	1.505491678	1.95E-05
1	2174395	26	downregulated	1.452700405	3.99E-08
1	2174395	27	upregulated	1.169556976	7.19E-06
1	2175279	23	downregulated	1.088981699	2.81E-08
1	2175279	24	downregulated	0.940540775	8.14E-07
1	2175279	25	upregulated	1.268586015	1.42E-10
1	2176369	20	downregulated	1.331409562	4.80E-24
1	2176369	30	upregulated	0.826247204	9.34E-11
1	2176775	26	downregulated	0.612744127	0.000462058
1	2176775	27	upregulated	0.835738618	1.33E-06
1	2176815	22	downregulated	0.761256778	0.002064163
1	2176815	27	upregulated	1.286008683	7.05E-08
1	2176841	28	downregulated	0.851904074	2.27E-06
1	2176841	29	upregulated	1.128472727	5.20E-10
1	2176843	26	downregulated	0.855782402	0.003252888
1	2176843	27	upregulated	0.725360807	0.015262955
1	2176981	22	downregulated	1.153732703	1.43E-07
1	2176981	25	upregulated	0.895098979	4.66E-05
1	2177486	26	downregulated	1.438199754	9.39E-19
1	2177486	27	upregulated	1.49575009	6.39E-20
1	2178183	27	downregulated	0.688683544	1.59E-18
1	2178183	28	upregulated	0.924682153	2.20E-31
1	2178185	25	downregulated	0.977073455	7.47E-11
1	2178185	26	upregulated	0.999620016	2.87E-11
1	2178203	26	downregulated	0.726787872	0.001000231
1	2178203	27	upregulated	1.027162245	1.62E-06
1	2178791	24	downregulated	1.147699537	4.11E-10

1	2178791	25	upregulated	1.050175601	8.47E-09
1	2178792	23	downregulated	1.832908206	1.34E-05
1	2178792	24	upregulated	1.506744571	0.000245081
1	2179098	25	downregulated	0.951522894	3.84E-11
1	2179098	26	upregulated	0.929929566	1.02E-10
1	2179466	22	downregulated	2.269618915	0.000108911
1	2179466	25	upregulated	2.117359167	0.000257652
1	2179602	25	downregulated	1.254837898	9.71E-14
1	2179602	27	upregulated	0.64590761	0.00014281
1	2179784	23	downregulated	1.156673664	3.63E-22
1	2179784	26	upregulated	1.355126504	9.52E-29
1	2180641	25	downregulated	0.948389764	1.99E-05
1	2180641	28	upregulated	0.600132292	0.010829253
1	2195957	23	downregulated	0.689953286	4.07E-07
1	2195957	29	upregulated	0.833721534	7.33E-10
1	2199433	20	downregulated	1.013929107	0.024792976
1	2199433	26	upregulated	1.287049281	0.002716503
1	2204904	26	downregulated	1.823011185	9.05E-11
1	2204904	27	upregulated	1.639542088	2.75E-09
1	2206429	24	downregulated	0.940384332	0.001656098
1	2206429	27	upregulated	1.508042859	3.54E-07
1	2208510	23	downregulated	1.437324556	2.70E-09
1	2208510	25	upregulated	0.79688782	0.00111251
1	2208632	25	downregulated	1.66743547	7.23E-31
1	2208632	26	upregulated	1.500801028	2.79E-26
1	2208689	27	downregulated	0.722431972	4.96E-32
1	2208689	28	upregulated	0.834011999	1.38E-41
1	2210009	24	downregulated	0.853818514	0.007291933
1	2210009	27	upregulated	0.926007398	0.003037701
1	2210181	28	downregulated	1.254380183	0.001533345
1	2210181	29	upregulated	1.271830186	0.001335229
1	2210309	25	downregulated	0.786296509	0.001084921
1	2210309	26	upregulated	0.775006289	0.001300369
1	2210463	22	downregulated	0.733083924	0.008663312
1	2210463	28	upregulated	1.091998628	2.75E-05
1	2210470	24	downregulated	0.728750978	0.000264915
1	2210470	28	upregulated	0.964944623	7.67E-07
1	2211799	24	downregulated	0.706762041	2.05E-26
1	2211799	27	upregulated	0.766211079	1.44E-30
1	2212311	25	downregulated	1.942273822	3.01E-111
1	2212311	26	upregulated	1.573901144	4.94E-81
1	2212312	24	downregulated	1.107787168	5.81E-13
1	2212312	25	upregulated	0.93106724	1.08E-09
1	2212331	24	downregulated	0.759341126	0.005313714
1	2212331	28	upregulated	1.767132394	1.21E-10
1	2212625	24	downregulated	0.996136404	5.32E-05
1	2212625	30	upregulated	0.618881615	0.023366917
1	2213867	23	downregulated	0.935252732	0.004558119
1	2213867	26	upregulated	1.276836548	6.08E-05
1	2214033	24	downregulated	0.92727682	0.000338244
1	2214033	25	upregulated	0.995941558	0.000108414
1	2214694	24	downregulated	1.52621132	5.61E-06
1	2214694	26	upregulated	1.367514985	3.93E-05
1	2214870	23	downregulated	0.856288252	0.039869942
1	2214870	24	downregulated	1.337908249	0.001764482
1	2214870	27	upregulated	1.238088505	0.004094864
1	2215215	27	downregulated	1.128135663	5.45E-05
1	2215215	28	upregulated	0.895053309	0.001479256
1	2215591	26	downregulated	0.762564998	0.000442333

1	2215591	27	upregulated	0.819311124	0.000157136
1	2215811	19	downregulated	1.001876042	0.000248607
1	2215811	26	upregulated	0.926498	0.000782905
1	2216104	25	downregulated	0.68926485	4.08E-25
1	2216104	26	upregulated	0.68810074	4.87E-25
1	2217040	23	downregulated	0.886292896	0.000192356
1	2217040	26	upregulated	1.076339058	4.72E-06
1	2225839	26	downregulated	0.832026599	4.46E-09
1	2225839	27	upregulated	0.735037777	2.29E-07
1	2225840	25	downregulated	1.322872603	3.14E-06
1	2225840	26	upregulated	1.441976831	4.60E-07
1	2225846	25	downregulated	1.362286154	3.07E-05
1	2225846	26	upregulated	1.187905548	0.000271669
1	2226262	25	downregulated	0.902705465	0.039102833
1	2226262	26	upregulated	0.899193321	0.040035361
1	2226794	24	downregulated	1.438007125	4.06E-22
1	2226794	27	upregulated	0.891327252	4.23E-10
1	2226797	22	downregulated	1.415309755	8.57E-33
1	2226797	25	upregulated	1.053618931	3.23E-20
1	2227889	24	downregulated	0.985081528	9.45E-08
1	2227889	26	upregulated	0.948791173	2.71E-07
1	2228512	28	downregulated	1.533512971	0.000178349
1	2228512	29	upregulated	1.012927328	0.017479045
1	2228841	25	downregulated	0.853666486	0.002504575
1	2228841	26	downregulated	0.654276825	0.016579664
1	2228841	27	downregulated	0.934618752	0.001788121
1	2228841	28	upregulated	1.730921643	1.14E-08
1	2316890	21	downregulated	0.72286974	0.000217247
1	2316890	26	upregulated	1.013489007	1.32E-07
1	2321348	23	downregulated	1.050447832	1.10E-07
1	2321348	29	upregulated	0.934328395	2.37E-06
1	2321349	27	downregulated	0.720477758	5.36E-12
1	2321349	29	upregulated	0.730298768	2.74E-12
1	2325231	27	downregulated	1.76635297	1.91E-11
1	2325231	28	upregulated	1.758945709	2.22E-11
1	2325232	26	downregulated	2.278852623	6.17E-21
1	2325232	27	upregulated	2.303877296	3.15E-21
1	2325233	25	downregulated	1.832235372	1.17E-33
1	2325233	26	upregulated	1.823666483	1.97E-33
1	2325234	24	downregulated	1.955304043	5.27E-06
1	2325234	25	upregulated	1.61453575	0.000102614
1	2326028	26	downregulated	1.382466207	7.54E-09
1	2326028	27	upregulated	1.070529106	6.06E-06
1	2326329	25	downregulated	1.091147248	5.22E-06
1	2326329	26	upregulated	0.671463123	0.008853944
1	2326746	22	downregulated	1.318604562	1.45E-09
1	2326746	26	upregulated	1.038869351	1.42E-06
1	2328563	23	downregulated	1.423097224	4.74E-06
1	2328563	25	downregulated	0.670602389	0.035412154
1	2328563	26	upregulated	1.257055572	5.12E-05
1	2329478	25	downregulated	0.890249323	0.001572162
1	2329478	26	upregulated	1.122782966	5.11E-05
1	2329479	26	downregulated	0.642976114	5.25E-12
1	2329479	27	upregulated	1.000628564	4.55E-26
1	2339402	25	downregulated	1.608243275	3.78E-06
1	2339402	27	upregulated	1.288955009	0.00013994
1	2339403	26	downregulated	1.912909652	0.000196521
1	2339403	28	upregulated	1.540925574	0.002169803
1	2343964	24	downregulated	1.004082003	3.68E-05

1	2343964	26	upregulated	0.832458166	0.000763083
1	2344426	26	downregulated	0.782535942	0.000540672
1	2344426	28	upregulated	1.306383702	7.99E-09
1	2344882	26	downregulated	0.928204594	2.25E-06
1	2344882	28	upregulated	0.710418934	0.000393806
1	2345184	26	downregulated	0.888882411	0.020779523
1	2345184	27	upregulated	1.186185136	0.001121963
1	2345224	25	downregulated	1.658522601	2.66E-13
1	2345224	27	upregulated	1.378539689	4.57E-10
1	2345359	23	downregulated	0.612314601	0.031320581
1	2345359	26	downregulated	1.413980914	7.56E-07
1	2345359	27	upregulated	1.688849377	7.43E-09
1	2346476	26	downregulated	0.714601304	0.00020541
1	2346476	27	upregulated	0.662996157	0.000673828
1	2346487	26	downregulated	2.061926501	1.57E-07
1	2346487	28	upregulated	1.06379227	0.006248494
1	2346903	22	downregulated	3.125155346	3.83E-11
1	2346903	25	upregulated	2.59619173	4.53E-09
1	2364925	24	downregulated	0.649850812	1.44E-26
1	2364925	26	upregulated	0.773251644	1.65E-36
1	2380537	24	downregulated	1.065359592	8.06E-16
1	2380537	25	upregulated	1.097220331	1.29E-16
2	21393685	23	downregulated	1.366055743	8.97E-05
2	21393685	25	upregulated	0.808756151	0.036951602
2	21394131	25	downregulated	1.862523115	0.00044247
2	21394131	26	upregulated	1.341613364	0.010900706
2	21394132	24	downregulated	2.483055753	2.41E-09
2	21394132	25	upregulated	1.128439138	0.003529547
2	21394939	26	downregulated	1.570382335	9.39E-14
2	21394939	29	upregulated	1.854778427	1.53E-17
2	21394969	24	downregulated	1.687414592	2.90E-12
2	21394969	27	upregulated	1.178535714	4.55E-07
2	21394972	25	downregulated	0.675533475	4.22E-34
2	21394972	28	upregulated	1.120410474	1.14E-84
2	21397264	25	downregulated	1.996042896	2.73E-105
2	21397264	29	upregulated	1.70793341	1.15E-83
2	21397266	23	downregulated	1.010332045	9.88E-06
2	21397266	27	upregulated	0.915480623	6.78E-05
2	21397267	22	downregulated	0.745469944	1.69E-15
2	21397267	26	upregulated	0.880866582	7.72E-21
2	21397268	26	downregulated	0.962457573	0.008345201
2	21397268	29	upregulated	0.964025196	0.008216731
2	21397646	25	downregulated	1.352980231	1.98E-18
2	21397646	26	upregulated	1.184067682	7.36E-15
2	21398798	24	downregulated	1.826401277	1.08E-35
2	21398798	27	upregulated	0.606454631	5.93E-06
2	21402726	26	downregulated	0.977020709	7.69E-28
2	21402726	27	upregulated	1.297828972	4.05E-45
2	21402785	19	downregulated	0.89528381	0.004416762
2	21402785	25	upregulated	0.937540248	0.002602431
2	21424208	21	downregulated	0.640165164	6.77E-07
2	21424208	26	upregulated	0.61221949	2.22E-06
2	21425085	24	downregulated	0.844762162	7.71E-06
2	21425085	28	upregulated	0.817116053	1.55E-05
2	21426193	26	downregulated	1.190315887	1.80E-43
2	21426193	27	upregulated	0.875965772	1.47E-25
2	21426202	25	downregulated	0.753033848	0.004709585
2	21426202	26	downregulated	0.635827502	0.012063305
2	21426202	27	upregulated	1.012263546	7.19E-05

5	20149000	25	downregulated	0.709318176	0.003473645
5	20149000	27	upregulated	0.684891371	0.005059162
5	20151190	21	downregulated	1.617052484	0.001453787
5	20151190	25	upregulated	1.709071047	0.000821732
5	20151355	26	downregulated	0.755727387	0.005194513
5	20151355	27	upregulated	1.267524435	1.25E-06
5	20173560	23	downregulated	0.948024698	5.83E-07
5	20173560	26	upregulated	1.163708106	1.09E-09
6	23289803	26	downregulated	1.708779148	3.63E-05
6	23289803	27	upregulated	0.859901374	0.014143918
6	23290528	24	downregulated	1.808134384	7.35E-07
6	23290528	27	upregulated	1.375497666	0.00010736
6	23290529	23	downregulated	3.175687303	3.33E-08
6	23290529	26	upregulated	1.482657547	0.002941766
6	23291782	25	downregulated	1.156328579	0.000522143
6	23291782	26	upregulated	1.184234457	0.000374654
6	23292726	23	downregulated	1.224974409	0.000300038
6	23292726	26	upregulated	1.110745456	0.00105477
6	23294140	25	downregulated	1.174279105	0.000175457
6	23294140	26	upregulated	1.050424232	0.000824615
6	23298106	23	downregulated	1.257244811	2.07E-06
6	23298106	27	upregulated	0.711005918	0.013136485
6	23298142	24	downregulated	2.81137444	3.24E-29
6	23298142	27	upregulated	1.852611496	5.73E-17
6	23298535	20	downregulated	1.957678954	0.001164432
6	23298535	25	upregulated	1.346520218	0.027616646
6	23298704	22	downregulated	0.911401106	0.000986472
6	23298704	23	downregulated	1.413617239	1.16E-06
6	23298704	24	upregulated	0.903345307	0.001097236
6	23298704	28	upregulated	0.954394401	0.001097236
6	23298820	22	downregulated	1.087082083	0.010933587
6	23298820	27	upregulated	1.40518662	0.00071981
6	23299198	23	downregulated	4.17217033	5.54E-23
6	23299198	26	upregulated	3.349756958	1.61E-18
6	23299340	25	downregulated	0.989460513	5.70E-07
6	23299340	27	upregulated	0.898134295	5.85E-06
6	23299758	26	downregulated	0.80126584	7.88E-06
6	23299758	27	upregulated	0.75455911	2.79E-05
6	23299914	24	downregulated	0.924236733	0.003819815
6	23299914	27	upregulated	1.145711986	0.000240438
6	23299921	24	downregulated	0.684081255	0.020080117
6	23299921	25	upregulated	0.751760445	0.009417386
6	23300407	23	downregulated	0.837265095	6.61E-07
6	23300407	26	upregulated	1.060210183	3.06E-10
6	23302842	21	downregulated	3.595741897	5.90E-15
6	23302842	24	upregulated	1.131346691	0.002414875
6	23304676	24	downregulated	1.649465977	3.41E-14
6	23304676	25	upregulated	1.487217511	3.50E-12
7	4025114	25	downregulated	1.538296395	7.26E-11
7	4025114	26	upregulated	0.983243292	2.13E-05
7	4027248	24	downregulated	1.519768075	1.23E-13
7	4027248	26	upregulated	0.849160526	2.14E-05
7	4027257	23	downregulated	1.218194643	9.30E-19
7	4027257	26	upregulated	1.381257758	5.54E-23
7	4027490	21	downregulated	0.823037219	0.000106426
7	4027490	28	upregulated	0.720539198	0.000868633
7	4027522	21	downregulated	1.980341305	9.96E-75
7	4027522	25	upregulated	1.345920446	2.48E-41
9	21772236	23	downregulated	1.89195534	0.001155994

9	21772236	24	upregulated	1.650602766	0.003839492
9	21804933	25	downregulated	0.933542222	0.007741916
9	21804933	26	upregulated	1.060715697	0.002099389
12	1408782	23	downregulated	2.112623963	2.10E-09
12	1408782	26	upregulated	1.217506038	0.000283603
12	1409486	26	downregulated	1.212081558	0.048257121
12	1409486	28	upregulated	1.345545463	0.02563309
12	1413144	23	downregulated	0.687756569	3.84E-06
12	1413144	24	upregulated	0.818253933	3.42E-08
13	2039115	24	downregulated	1.663599182	4.67E-11
13	2039115	26	upregulated	1.067178682	1.13E-05
13	2051729	24	downregulated	0.766666239	0.000107984
13	2051729	28	upregulated	0.766532991	0.000108309
13	2096600	24	downregulated	1.780047527	1.26E-07
13	2096600	27	upregulated	1.528331681	3.38E-06
13	2136667	26	downregulated	1.267685559	8.04E-19
13	2136667	27	upregulated	1.173434268	1.32E-16
15	269211	23	downregulated	1.319902316	0.001747524
15	269211	26	upregulated	1.183140029	0.005311224
15	296868	23	downregulated	0.849387044	0.005379274
15	296868	24	downregulated	0.656436668	0.022477552
15	296868	27	upregulated	0.900206654	0.002920819
16	2074719	26	downregulated	1.058635574	4.93E-08
16	2074719	27	upregulated	1.394749409	1.71E-12
16	2089279	21	downregulated	0.702952243	0.00034532
16	2089279	25	upregulated	1.176092618	9.09E-10
16	2090319	26	downregulated	0.718406264	1.44E-05
16	2090319	27	upregulated	0.853017678	2.56E-07
16	2095941	24	downregulated	2.308673821	3.36E-30
16	2095941	26	upregulated	1.543850568	3.59E-17
16	2097217	25	downregulated	1.054288405	0.016622197
16	2097217	27	upregulated	1.079922353	0.013732821
16	2097814	25	downregulated	0.778975307	0.010584004
16	2097814	27	upregulated	0.742683769	0.01631692
16	2097990	27	downregulated	0.889447999	0.002048234
16	2097990	28	upregulated	1.058422571	0.00020206
16	2101377	22	downregulated	0.910207946	8.55E-10
16	2101377	24	upregulated	0.707711202	2.07E-06
20	835296	24	downregulated	2.318505654	3.28E-06
20	835296	27	upregulated	2.226310271	6.47E-06
21	2830779	24	downregulated	1.228750452	8.09E-07
21	2830779	25	upregulated	0.7235512	0.001930251
23	22406617	25	downregulated	0.939351556	0.000452207
23	22406617	27	upregulated	0.733156202	0.008160909
24	195696	25	downregulated	0.74721662	7.77E-09
24	195696	26	upregulated	0.816331947	2.78E-10
25	24113257	25	downregulated	0.975027005	2.72E-06
25	24113257	26	upregulated	0.817746268	9.04E-05
27	20106745	24	downregulated	0.856661203	1.35E-08
27	20106745	25	upregulated	0.844075811	2.19E-08
27	20110252	22	downregulated	0.784585849	1.32E-11
27	20110252	29	upregulated	1.118873428	1.97E-21
27	20112394	22	downregulated	0.986414274	0.002885425
27	20112394	25	upregulated	0.896451459	0.007726722
27	20113437	23	downregulated	0.659514853	1.74E-06
27	20113437	29	upregulated	0.651637453	2.36E-06
27	20113892	24	downregulated	0.720925696	2.32E-06
27	20113892	27	upregulated	1.055345725	4.01E-12
27	20114078	24	downregulated	0.822327361	6.91E-27

27	20114078	25	upregulated	0.839362372	6.94E-28
28	117210	25	downregulated	0.605635408	0.002591425
28	117210	26	upregulated	0.836700897	1.88E-05
28	186610	23	downregulated	0.830744816	8.50E-17
28	186610	24	upregulated	0.703971701	1.44E-12
31	22518317	25	downregulated	1.671252541	7.99E-77
31	22518317	26	upregulated	1.580234073	1.79E-70
31	22520484	22	downregulated	0.966177601	0.012713775
31	22520484	25	upregulated	1.066709132	0.004972078
34	20522	22	downregulated	0.722203788	0.014993436
34	20522	23	downregulated	1.18181259	0.0001575
34	20522	27	downregulated	0.898135665	0.003071368
34	20522	28	upregulated	1.37991774	9.62E-06
35	161194	26	downregulated	0.759204441	4.87E-38
35	161194	27	upregulated	1.000618175	2.37E-62
37	1669194	23	downregulated	1.118090845	0.001123544
37	1669194	27	upregulated	1.084980245	0.001614953
37	1675657	24	downregulated	1.226833667	3.78E-32
37	1675657	27	upregulated	0.950373756	7.51E-21
38	2239376	23	downregulated	2.389264982	2.78E-08
38	2239376	26	upregulated	1.991238471	1.29E-06
42	10050	21	downregulated	1.172989485	0.012043988
42	10050	22	upregulated	1.267249563	0.006674048
44	368732	24	downregulated	1.94603211	2.52E-05
44	368732	26	upregulated	1.083792983	0.024157848
44	368914	22	downregulated	0.91928307	0.038803114
44	368914	24	downregulated	1.241655047	0.006885569
44	368914	25	downregulated	1.507220391	0.002048389
44	368914	28	upregulated	1.519247079	0.001875382
49	1789945	27	downregulated	0.610924569	8.90E-09
49	1789945	28	upregulated	0.719346657	1.10E-11
50	631501	23	downregulated	0.949593311	0.000169971
50	631501	26	upregulated	1.146121949	4.82E-06
50	642917	24	downregulated	0.888556381	1.47E-05
50	642917	25	upregulated	0.604623244	0.003100267
51	22973226	27	downregulated	0.612630319	1.22E-05
51	22973226	28	upregulated	1.024571048	1.76E-13
52	2090448	24	downregulated	1.431630766	3.95E-12
52	2090448	27	upregulated	0.999205219	6.99E-07
53	549429	23	downregulated	0.834686933	0.004219285
53	549429	25	upregulated	1.028140985	0.000341335
54	2454905	25	downregulated	0.996605139	2.10E-05
54	2454905	26	upregulated	0.964931053	3.88E-05
54	2470735	23	downregulated	1.17631231	0.000208814
54	2470735	26	upregulated	1.106272183	0.000513732
54	2470736	24	downregulated	1.792204094	0.002419583
54	2470736	27	upregulated	1.244654526	0.040169296
54	2474023	27	downregulated	0.670608971	0.000272562
54	2474023	28	upregulated	0.790775242	1.86E-05
55	23631984	26	downregulated	1.802301794	2.84E-126
55	23631984	27	upregulated	1.458903	5.63E-91
59	14335	24	downregulated	0.63827515	0.022181998
59	14335	28	upregulated	0.855325701	0.000969887
60	129222	23	downregulated	1.224327906	0.00021729
60	129222	27	upregulated	2.024748341	5.72E-09
62	22276358	24	downregulated	1.26378448	0.004914706
62	22276358	27	upregulated	0.992882706	0.030499254
67	904602	26	downregulated	1.08748938	0.004685333
67	904602	28	upregulated	0.99611649	0.010897956

68	3535176	23	downregulated	0.849651174	0.010992816
68	3535176	26	upregulated	0.824691614	0.014467077
68	3535325	23	downregulated	0.842968883	4.07E-05
68	3535325	24	downregulated	0.775257952	9.01E-05
68	3535325	26	upregulated	0.780686442	8.02E-05
68	3535325	27	upregulated	1.076651687	1.38E-07
70	1217116	24	downregulated	1.395118652	8.98E-16
70	1217116	25	upregulated	1.193024645	2.62E-12
72	1878839	23	downregulated	1.335092334	0.010969304
72	1878839	24	upregulated	1.411473912	0.007453188
72	1878841	25	downregulated	2.131567097	0.003053047
72	1878841	26	upregulated	1.352887469	0.044738256
72	1883079	23	downregulated	1.010755789	0.000216515
72	1883079	25	upregulated	0.880776352	0.001532509
72	1885227	23	downregulated	1.707273716	5.71E-16
72	1885227	27	upregulated	1.535275278	1.21E-13
72	1885773	24	downregulated	1.418021378	3.60E-09
72	1885773	29	upregulated	0.604159171	0.013904241
73	872503	25	downregulated	1.126258492	0.001864642
73	872503	26	downregulated	0.68251455	0.045703783
73	872503	27	upregulated	1.385161684	0.000134156
73	872524	23	downregulated	1.0834538	0.034740044
73	872524	24	upregulated	1.98521313	0.000147201
75	24255481	26	downregulated	0.986463696	7.22E-07
75	24255481	29	upregulated	0.824190813	3.50E-05
76	77590	22	downregulated	2.175525031	0.001732967
76	77590	25	upregulated	1.936248307	0.004724204
88	1687559	24	downregulated	1.45201389	0.000527457
88	1687559	27	upregulated	1.147523798	0.006896023
88	1706992	24	downregulated	1.812118103	3.43E-11
88	1706992	28	upregulated	0.870614114	0.001116941
89	842419	24	downregulated	1.310044904	0.000630916
89	842419	25	upregulated	0.912908768	0.01927924
96	2179896	24	downregulated	1.154875977	0.000139946
96	2179896	27	upregulated	0.988091787	0.001247902
97	21143634	23	downregulated	0.952397587	1.12E-05
97	21143634	24	downregulated	0.762776624	0.000278295
97	21143634	27	upregulated	1.315465874	1.90E-09
100	2200505	24	downregulated	3.940438457	3.12E-38
100	2200505	27	upregulated	3.076468246	1.44E-29
118	6216835	25	downregulated	2.824874939	2.98E-09
118	6216835	26	upregulated	1.500695406	0.000344595
118	6217209	25	downregulated	0.648337395	0.001356742
118	6217209	26	upregulated	0.785699389	7.00E-05
131	1180963	24	downregulated	0.772322531	0.047769541
131	1180963	25	downregulated	1.372735874	0.000469675
131	1180963	27	upregulated	1.225902494	0.001924062

Column 1: piRNA IDs from Brennecke et al. 2007
Column 2: start position in reference genome version dm3 (BDGP Release 5)
Columns 2 and 3: significant based on FDR <=0.05 and Cohen's d >= 0.6.
