# Supplementary material for: The exonuclease Nibbler regulates age-associated traits and modulates piRNA length in Drosophila
Source: Aging Cell. 2015 Mar 6;14(3):443–52. doi: 10.1111/acel.12323 (PMC4406673; doi:10.1111/acel.12323)
Supplement: Supplementary file 2 [file acel0014-0443-sd2.docx]

***Supplemental Material for:***

**The exonuclease Nibbler regulates**

**age-associated traits AND MODULATES**

**piRNA length in *Drosophila***

Virzhiniya L. Feltzin^1,$^, Mugdha Khaladkar^1,2,$^, Masashi Abe^1,$^ ,

Michael Parisi^1^, Gert-Jan Hendriks^1*^,

Junhyong Kim^1,2^, and Nancy M. Bonini^1^^.

**^1^Department of Biology and the ^2^Penn Genome Frontiers Institute**

**University of Pennsylvania, Philadelphia, PA 19104, USA**

**^$^ These authors contributed equally and should be considered co-first authors.**

* Current address: Friedrich Miescher Institute for Biomedical Research, Maulbeerstrasse 66, 4058 Basel, Switzerland

**^**author for correspondence: [nbonini@sas.upenn.edu](mailto:nbonini@sas.upenn.edu)

**
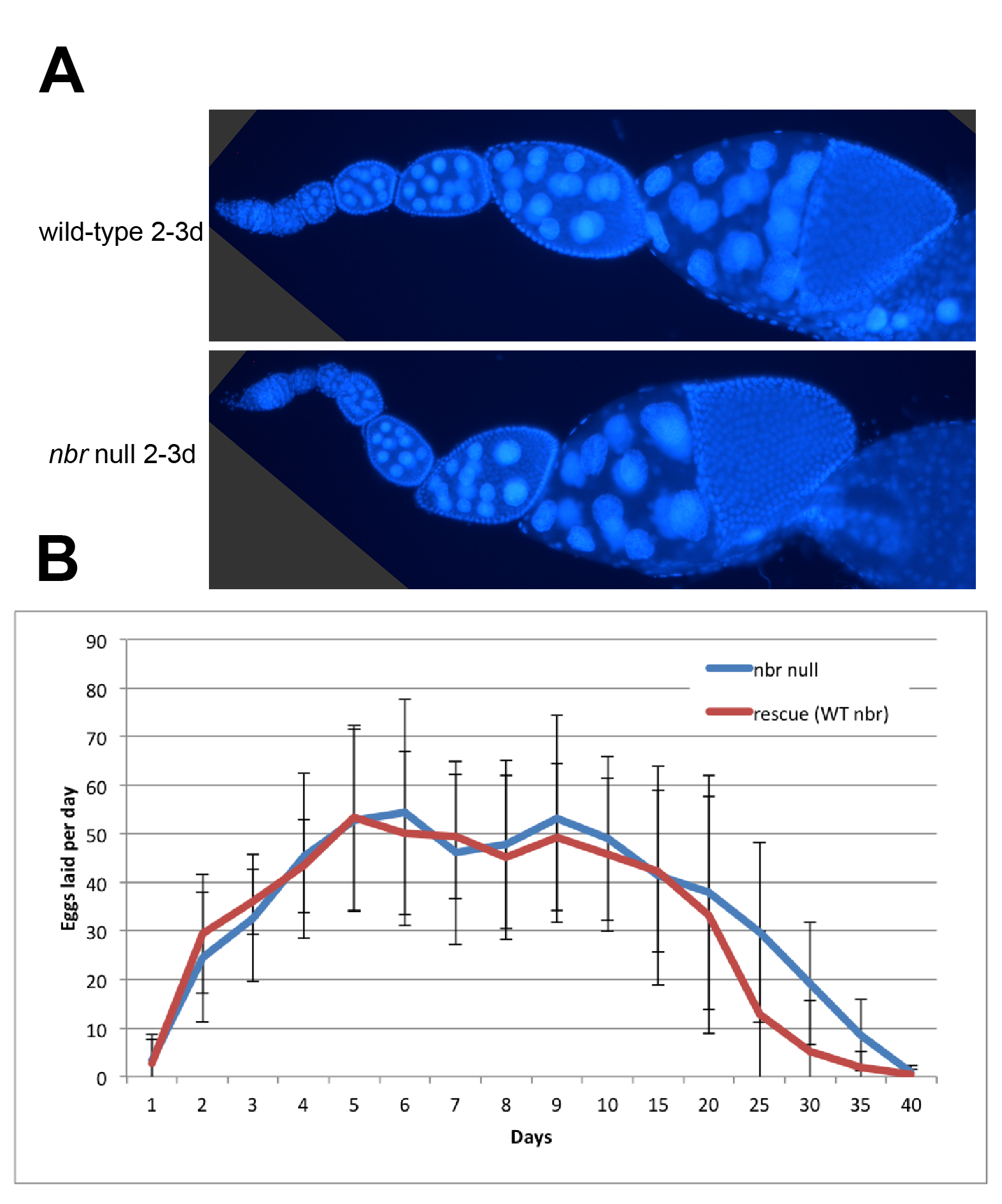
**

**Figure S1. Nbr null females have normal ovaries and normal fecundity with age.**

A. Hoechst staining of whole mount preparations of ovarioles from wild-type and *nbr* null (*nbr*^f02257^/*Df(2L)BSC312*), from 2~3d female flies. The ovaries of *nbr* null animals look normal. B. Count of eggs laid per day by *nbr* null (*nbr*^f02257^/*Df(2L)BSC312*) and *nbr* wild-type rescue (*nbr*^f02257^/*Df(2L)BSC312*; pCaSper-*nbr*) females. Loss of function of *nbr* has no effect on female egg laying initially or with age. For *nbr* null females, n=14; for *nbr* wild-type rescue females, n=15.

**
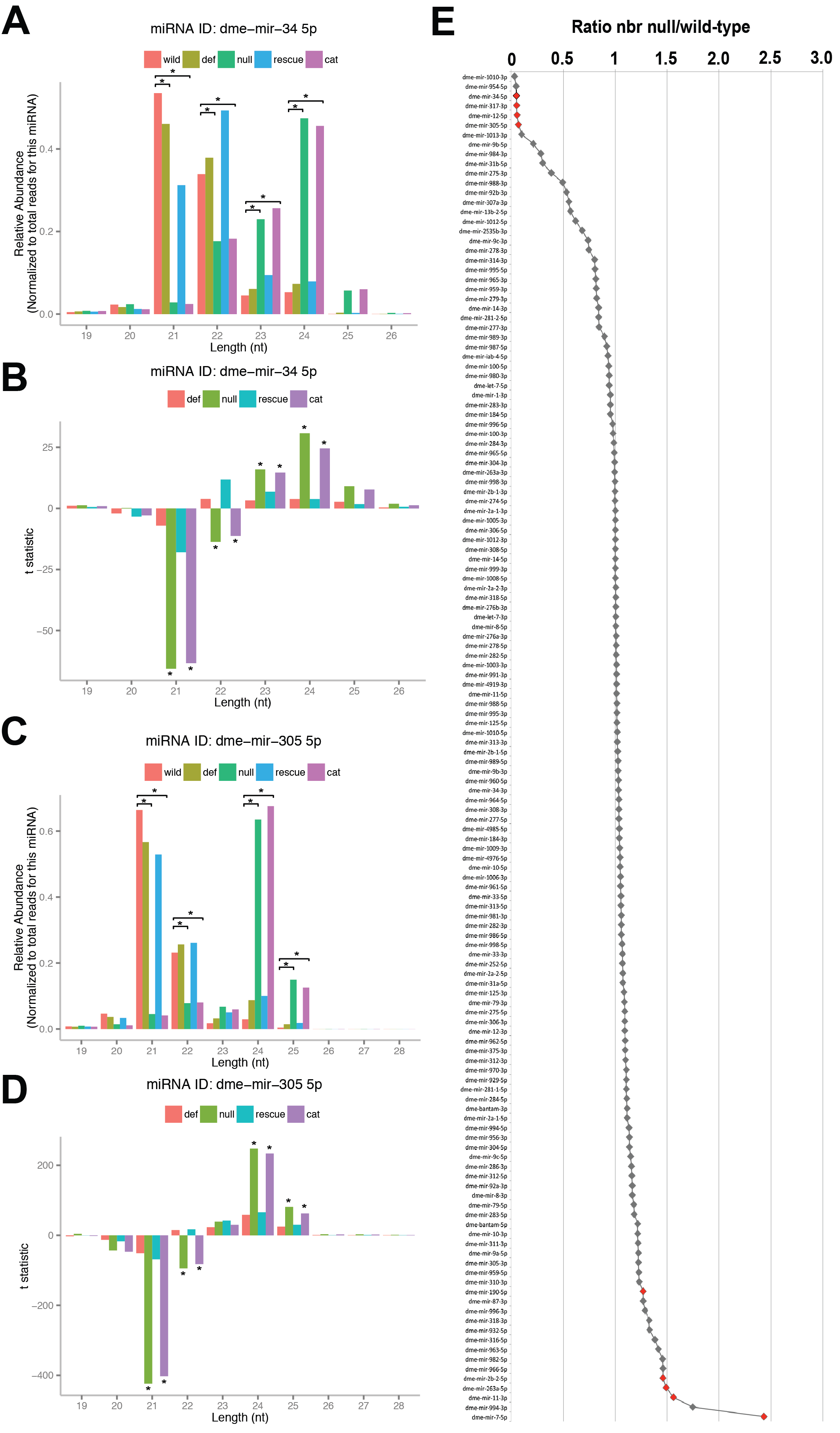
**

**Figure S2. Length distribution of select miRNAs in ovary deep-sequencing libraries.** A,B. Abundance of different length isoforms for the miRNAs miR-34-5p and miR-305-5p relative to the total number of reads for that miRNA in the five studied genotypes. C,D. t-statistic describing the ratio of the departure in the abundance of different length isoforms of miR-34-5p and miR-305-5p in ovaries of experimental genotype flies compared to wild-type ovaries. The t-statistic was converted to the p-value in the same manner as in Figure 5. E. Plot of miRNA length ratios for *nbr* null (*nbr*^f02257^/*Df(2L)BSC312*) compared to wild-type ovaries. For each miRNA, the ratio of the abundance of the most frequent length isoform to that of all other isoforms starting with the same 5' nt position was calculated in wild-type and *nbr* null. The ratio in *nbr* null was divided by the ratio in wild-type to generate the "Ratio *nbr* null/wild-type" value as shown on the Y-axis. miRNAs with a high or low null/wild-type ratio are those whose isoform distribution is predicted to be affected in *nbr* null compared to wild-type. Red diamonds highlight those miRNAs that were experimentally verified as affected in a previous study of *nbr* (Liu et al. 2011). We note that the ratio for each miRNA may be different than the ratio reported in the previous Liu et al (2011) study, due to a different precise miRNA length isoform being most abundant in the respective libraries. The graph is intended to highlight potential Nbr-dependent miRNAs, which are those with extreme ratios. Genotypes tested: wild: wild-type (w^118^) def: *nbr* deficiency/+ (*Df(3L)BSC312*/+), null: *nbr* null (*nbr^f02257^/Df(3L)BSC312*)*,* rescue: rescue with wildtype *nbr* (*nbr* null; pCaSper-*nbr* (wild-type)), and cat: rescue with catalytically-dead *nbr* (*nbr* null; pCaSper-*nbr* (D435A,E437A)). *p<0.01 compared to wild-type control.
